# Individual/Peak Gamma Frequency: What Do We Know?

**DOI:** 10.3390/brainsci13050792

**Published:** 2023-05-12

**Authors:** Aurimas Mockevičius, Kristina Šveistytė, Inga Griškova-Bulanova

**Affiliations:** Institute of Biosciences, Life Sciences Centre, Vilnius University, Saulėtekio av. 7, LT-10257 Vilnius, Lithuania

**Keywords:** individual gamma frequency, peak gamma frequency, functional aspects

## Abstract

In recent years, the concept of individualized measures of electroencephalographic (EEG) activity has emerged. Gamma-band activity plays an important role in many sensory and cognitive processes. Thus, peak frequency in the gamma range has received considerable attention. However, peak or individual gamma frequency (IGF) is rarely used as a primary measure of interest; consequently, little is known about its nature and functional significance. With this review, we attempt to comprehensively overview available information on the functional properties of peak gamma frequency, addressing its relationship with certain processes and/or modulation by various factors. Here, we show that IGFs seem to be related to various endogenous and exogenous factors. Broad functional aspects that are related to IGF might point to the differences in underlying mechanisms. Therefore, research utilizing different types of stimulation for IGF estimation and covering several functional aspects in the same population is required. Moreover, IGFs span a wide range of frequencies (30–100 Hz). This could be partly due to the variability of methods used to extract the measures of IGF. In order to overcome this issue, further studies aiming at the optimization of IGF extraction would be greatly beneficial.

## 1. Introduction

In recent years, the concept of individualized measures of electroencephalographic (EEG) activity has emerged. For example, peak alpha frequencies or peak theta frequencies are frequently assessed in various conditions and are used as markers for certain processes to track the effect of behavioral manipulations or neurostimulation [1,2,3]. Similarly, interest in peak frequency in the gamma band is increasing [4,5,6,7,8,9].

Gamma oscillations can be measured during the resting-state condition [10], in response to stimulation of basically any sensory modality [11,12,13] and while performing cognitive tasks [14]. However, during the resting state, no prominent peak in the gamma range is observed and it is technically difficult to extract the information [15]. Thus, methods for the estimation/extraction of peak frequencies are being developed and tested [8,16,17]. Nevertheless, it is still a question whether there is a relationship between peak gamma frequency as evoked in response to a certain stimulus or task and the individual gamma frequency (IGF) indexing individual-specific resonant properties of the brain. Indeed, Zaehle et al. [17] demonstrated a correlation between peak gamma frequencies estimated using an auditory steady-state response approach and those extracted from evoked responses to brief auditory stimuli, suggesting that both types of responses might reflect overlapping processes. However, little is known about the nature and functional significance of individual or peak gamma frequency. With this review, we attempt to comprehensively overview available information on the functional properties of peak gamma frequency addressing its relationship with various processes and/or modulation by various factors.

To note, the terminology used to address the phenomenon is highly diverse. While some studies use the terms “peak gamma frequency” [18] or “gamma peak frequency” [19], in others, the term “individual gamma frequency” [7] can be found. All of these terms generally denote a specific individual frequency in the gamma band (mostly 30–80 Hz) that is characterized by the highest amplitude, power or phase-locking values. Thus, in the present review, the terms “peak gamma frequency” and “individual gamma frequency” are used interchangeably.

## 2. Literature Search

To make the inclusion as wide as possible, two searches were performed using the following terms: peak-gamma-frequency OR gamma-peak-frequency OR individual-gamma-frequency OR peak-gamma-band OR individual-gamma-band. First, a search in PubMed was performed, resulting in a total of 32 entries. Then an additional search was carried out in Google Scholar, which yielded 964 entries in total. Duplicates, abstracts and review papers were excluded. Titles and abstracts of 847 original articles in English were screened. Specifically, we included only the papers in which (a) individual/peak gamma frequency was estimated and (b) the relationship of individual/peak gamma frequency with certain factors was tested. If the information in the abstract was insufficient, the whole article was viewed. This resulted in 94 papers being mentioned in this review. As the literature search was not conducted on a systematic basis, the evidence reviewed may be not exhaustive.

## 3. Methodological Aspects

A systematic review of the search results was not possible due to the fact that IGF is rarely identified as a primary measure of interest and is most frequently reported as a side measure. Furthermore, studies that were selected after the search were characterized by a high heterogeneity of approaches that were used to estimate peak gamma frequencies. In human research, a few studies estimated peak gamma frequency from resting-state EEG activity [20,21]. Others extracted IGF from gamma activity recorded with EEG or magnetoencephalography (MEG) during visual [22,23], auditory [24] or somatosensory [25,26] stimulation or while performing motor [27,28] or cognitive [29,30] tasks. Finally, some authors specifically tested the maximal response range using periodic stimulation [9,31]. A number of animal studies also investigated peak gamma frequency employing electrocorticography (ECoG) during sensory stimulation [32,33] as well as motor [34,35] or cognitive [36,37] activity. In addition, IGF was also extracted from in vitro recordings after inducing gamma activity in brain slices using various substances [38,39]. Depending on the type of stimulation used and its parameters, gamma peaks were estimated from the recordings of different brain areas and in both low gamma (~30–60 Hz) and high gamma (~60–100 Hz) ranges. Despite the high variety of methods and approaches used, for the ease of comprehension, the selected studies were grouped by the factors that demonstrated an association with IGF.

## 4. Endogenous Determinants of Peak Gamma Frequency

Peak gamma frequency appears to be a relatively stable characteristic of brain functioning and tends to show high consistency within a subject. Tan et al. [40] demonstrated the stability of visually induced peak gamma frequencies across repeated measurements within a subject. High test–retest consistency of auditory-evoked IGF was also reported [41]. Moreover, van Pelt et al. [42] suggested the genetic determination of the gamma-band peak frequency to be around 91%. In their study, visually induced peak gamma frequencies were highly correlated in monozygotic twins, whereas no correlation was found in dizygotic twins or unrelated subjects. Yet, certain endogenous factors were shown to contribute to the individual stability and variability of IGFs. Among those, anatomical features of the brain, neurochemical balance in normal and diseased conditions and the state of the subject can be highlighted.

### 4.1. Anatomical Features

The size of the neural networks is known to determine the frequency of the network operation [43]. Certain anatomical factors, including the surface area or volume of the visual cortex [44,45,46] and cuneus [47], were shown to be positively related to visually evoked/induced peak gamma frequencies (Table 1). In line with this finding, lesions of the primary visual cortex in monkeys resulted in decreased IGFs in the extrastriate cortex [48]. In contrast, a negative correlation of gamma peak frequency with the occipital cortex surface area and pericalcarine volume was demonstrated [23]. Although peak frequencies within the gamma range seem to be unrelated to the cortical thickness of the primary visual cortex [22,23,46,49], a positive relationship was shown for the pericalcarine [50,51], the cuneus [47] or the whole occipital cortex [23]. In addition, higher IGF in the presence of higher white matter density within the corpus callosum [52] and gray matter thickness in the occipital cortex [23] were reported. For the somatosensory gamma activity, the thickness of the primary somatosensory cortex was inversely related to peak gamma frequency [26]. On the contrary, some studies failed to detect correlations between IGF and various cortical measures (e.g., [22,46,49]); however, it should be noted that these experiments were performed in relatively small samples (12–34 subjects) and could have been underpowered to detect relevant associations.

### 4.2. Neurochemical Balance

Another endogenous aspect that was shown to be related to peak gamma frequency is the balance of neurotransmitters. The generation of gamma oscillations is based on the balance of excitation and inhibition [53,54]. Rapid adjustments in the inhibition result in changes in the oscillation interval and in the frequency of oscillation [55,56]. Consequently, most studies addressed the association between the concentration of GABA and peak gamma frequency (Table 2). However, the results are not conclusive as both positive relationship between IGF and GABA concentration levels [29,57,58,59] and no relationship [22,24,51,60] were shown. Again, it is possible that small sample sizes (14–50 subjects) could have hindered the ability to reveal potential relationships. To note, a positive association between GABA_A_ receptor density in the primary visual cortex and visually induced gamma frequency [61] and an inverse relationship between IGF and glutamate concentration in the lateral occipital cortex [30] were also observed. However, other studies did not find a significant correlation between IGF and glutamate [24,60].

Importantly, manipulation of the activity of neurotransmission by psychoactive substances showed various effects on peak gamma frequencies. IGF was reported to decrease in the visual cortex after administering alcohol [62], ketamine [63], lorazepam [64] or GABA reuptake inhibitor tiagabine [65]. Similarly, dopaminergic D4 receptor agonist A-412997 [66] and apomorphine [67] injection in rats or cannabinoid receptor agonist arachidonylcyclopropylamide injection into entorhinal cortex slices [38] reduced IGF. Conversely, kainate [68], carbachol and (RS)-3,5-dihydroxyphenylglycine [39], levodopa [67] and cannabinoid receptor antagonist LY320135 [38] were all found to increase peak frequencies in the gamma range.

### 4.3. Neuropsychiatric Disorders

It is well known that both neuroanatomical changes and neurotransmitter imbalances are linked with various pathological conditions, e.g., schizophrenia [69], depression [70] and neurodegenerative disorders [71]. Hence, peak gamma frequencies have been shown to be altered in neuropsychiatric disorders (Table 3). A few studies reported that patients with schizophrenia display lower IGFs compared to controls [9,29]. In addition, lower peak gamma frequencies in Alzheimer’s disease [21] and dyslexia [5] patients compared to controls were reported. On the contrary, an increase in peak gamma frequency was observed in people with autistic traits and autism spectrum disorder [18,72]. Finally, no IGF difference was found between healthy controls and patients with photosensitive epilepsy [73], visual snow syndrome [74] or schizoaffective bipolar disorder [75]. A relationship between phenomenological symptoms and cognitive abilities was also demonstrated: Subjects with schizophrenia reporting higher self-disorder scores had lower parietal peak gamma frequencies in response to proprioceptive stimulation [76], similar to multiple sclerosis patients with cognitive impairment who showed lower IGFs compared to patients with retained cognitive functions and controls [77].

### 4.4. Brain States

Peak frequencies in the gamma range were shown to depend on the subjects’ general state (Table 4). For example, peak gamma frequencies detected in response to auditory stimulation were shown to decrease with higher doses of isoflurane [78] and propofol [79] sedation. Similarly, lower IGF was shown in anesthetized monkeys compared to their awake state [80]. In contrast, Saxena et al. [81] found no differences in visually induced IGF between awake and propofol-induced sedation states. Lozano-Montes et al. [34] demonstrated that peak gamma frequency was higher in rats during quiet wakefulness than during self-grooming behaviors. Moreover, peak gamma frequencies were shown to depend on the state of the female organism: During the luteal phase, peak gamma frequencies were significantly higher compared to the peak frequencies in the follicular phase during the menstrual cycle [82].

Finally, as neuromodulational techniques are increasingly used to change the state of the brain, the effect of neuromodulation on peak gamma frequencies was tested. However, the knowledge is still limited: Transcranial alternating current stimulation (tACS) was reported to increase IGF [4,83,84], whereas transcranial direct current stimulation (tDCS) did not modulate IGF [85,86,87]. In addition, non-invasive vagal nerve stimulation was shown to decrease IGF [88]. The abovementioned suggests the potential of gamma peak frequencies to be modulated.

**Table 4:** Selected studies that reported IGF’s relationship with brain states.

Author and Year	Sample	Methods	IGF Measure	Relevant Findings
Baltus et al., 2018 [4]	26 healthy adults	EEG, tACS, auditory steady-state stimulation, gap detection task	Amplitude	IGF: 49 Hz, ~37–63. In IGF + 4 Hz stimulation group, IGF increased after tACS; in IGF-4 Hz group, IGF decreased after tACS
Lozano-Montes et al., 2020 [34]	29 Long Evans rats	In vivo LFP, optical stimulation, deep brain stimulation, behavioral tests	Power	IGF during quiet wakefulness: ~53 Hz, ~50–58 Hz; IGF during self-grooming: ~50 Hz, ~45–53 Hz
Munglani et al. 1993 [78]	7 healthy adults	EEG, isoflurane, auditory click stimulation, cognitive tests	Power	IGF awake: 32.8 Hz, 28–41 Hz. IGF anesthetized: 24.8 Hz, 21.5–30.6 Hz
Andrade et al. 1996 [79]	12 healthy adults	EEG, propofol, auditory click stimulation, cognitive assessment	Power	IGF: 37.6 Hz, 33.5–41.5 Hz. IGF in light sedation: 29.9 Hz, 23.5–35.5 Hz; IGF in deep sedation: 27.1 Hz, 20.5–35.5 Hz
Xing et al., 2012 [80]	2 monkeys	In vivo LFP, anesthesia, visual gratings	Amplitude	IGF in awake: 60 ± 9 Hz, ~55–64 Hz; IGF in anesthetized: 40 ± 8.8 Hz, ~35–42 Hz
Saxena et al., 2013 [81]	15 healthy adults	MEG, propofol, visual gratings	Amplitude	IGF: ~57 Hz. No difference in IGF between awake and sedated states
Sumner et al., 2018 [82]	20 healthy female adults	EEG, blood tests, visual gratings	Power	IGF depends on menstrual phase. For moving stimuli, IGF in luteal phase: 63.42 ± 5.3 Hz; IGF in follicular phase: 59.86 ± 7.19 Hz. For stationary stimuli, IGF in luteal phase: 58.16 ± 3.95 Hz; IGF in follicular phase 52.41 ± 3 Hz. No correlations between IGF and hormone levels
Baltus et al., 2020 [83]	16 healthy adults	EEG, tACS, auditory steady-state stimulation, gap detection task	Amplitude	IGF: ~35–60 Hz. Negative correlation of IGF with gap detection threshold (rho = −0.6). Positive correlation of IGF with change in performance after tACS in the experimental group (rho = 0.81), but not in the control group
Rufener et al., 2022 [84]	30 children with developmental dyslexia (DD)	EEG, tACS, auditory steady-state stimulation, language assessment	Power	Before tACS application, IGF in tACS group: 40.28 ± 6.22 Hz; IGF in controls: 41.07 ± 6.04 Hz. Immediate effects, ΔIGF in tACS group: 3.38 ± 1.89 Hz; ΔIGF in controls: 1.08 ± 1.85 Hz. Long-term effects, ΔIGF in tACS group: 3.91 ± 1.48 Hz; ΔIGF in controls: −1.58 ± 1.13 Hz
Dawood et al., 2022 [85]	49 healthy adults	EEG, tDCS, checkerboard visual stimulus	Power	No difference in IGF between pre- and post-tDCS
Wilson et al., 2017 [87]	35 healthy adults	MEG, MRI, tDCS, visual gratings	Amplitude	tDCS did not modulate IGF
Lewine et al., 2019 [88]	8 healthy adults	EEG, auditory stimulation, non-invasive vagal nerve stimulation	Power	Baseline IGF at Oz electrode: 44–49 Hz. IGF post-stimulation decreased by 3–4 Hz

Abbreviations: EEG—electroencephalogram; IGF—individual gamma frequency; LFP—local field potential; MEG—magnetoencephalogram; MRI—magnetic resonance imaging; tACS—transcranial alternating current stimulation; tDCS—transcranial direct current stimulation.

### 4.5. Age

Lastly, age is regarded as one of the factors modulating peak gamma frequency (Table 5). Significant reductions in gamma peak frequencies were observed in the medial frontal cortex of aged rats, but not in the hippocampus [37]. In humans, the slowing of IGF in the primary visual cortex [22,23,47,50,89,90,91,92,93,94] and primary motor cortex [59]⁠ with age was observed. Similarly, Purcell et al. [31] demonstrated an age-related decrease in peak gamma frequencies obtained from the auditory envelope following responses within the 30–50 Hz range. While many studies report a negative relationship between IGF and age, Duygun et al. [20] showed that peak gamma frequency tends to increase from childhood to young adulthood; however, it decreases with age in the adult population. In contrast, Poulsen et al. [95,96] reported an increase in IGF with age, whereas a few studies [24,46,51] showed no significant relationship between gamma peak frequencies and age, potentially due to testing subjects of relatively narrow age ranges (see Table 5). However, Proskovec et al. [26] also failed to detect a significant link between age and IGF, despite including subjects of a wide range of ages.

## 5. Peak Gamma Activity in Response to Sensory Stimulation

Gamma activity is generated in response to a stimulus of any modality [11,12,13]. Accumulating evidence suggests that various sensory processes have an effect on IGF.

### 5.1. Visual Processing

So far, research on peak frequency is mostly focused on visually evoked and induced gamma responses. It seems that depending on the type of a visual stimulus, IGF can be affected differently (Table 6). Higher IGFs are obtained with increasing stimulus contrast [23,33,36,73,97,98,99,100,101], velocity [23,91,92,102,103,104] and in the presence of orthogonal masks [99]. An increase in IGF was also observed for preferred stimulus orientation [103] and grated stimulus discontinuity [104] in monkeys. In contrast, peak gamma frequencies are inversely related to the size of a visual stimulus [19,32,98,105] and grating [32]. Yet, some studies showed that the size of a visual stimulus has no effect on peak gamma frequency [44,59]. Several studies provided contradictory results regarding visual stimulus eccentricity: Lima et al. [33] and van Pelt and Fries [19] reported an inverse relationship between eccentricity and IGF, while Gregory et al. [44] observed an increase in IGF in the presence of higher eccentricity. A biphasic pattern of gamma peak frequency modulation in the early visual cortex related to the repetition of the same visual stimulus was demonstrated in both humans [106] and monkeys [107]: IGF remained unchanged in humans and decreased in monkeys during the initial repetitions of a stimulus, but further repetitions resulted in a steady increase in IGF, possibly reflecting adaptive neuronal processing of recurring stimuli. In general, some studies suggest that individual system properties as well as the moment of assessment might affect the results of IGF estimation. For example, Kahlbrock et al. [89] reported higher IGF in individuals with higher critical flicker frequency, whereas Brunet et al. [19] observed a significantly decreased peak gamma frequency immediately after saccades in monkeys.

### 5.2. Auditory Processing

Research in the auditory domain has demonstrated a relationship between IGF and auditory perception (Table 7). Multiple studies showed that higher IGFs are related to better performance in the gap detection task [31,41,83], which is commonly used as a measure of temporal auditory acuity. Moreover, peak gamma frequency was significantly related to the maximum perceptible modulation frequency [31]. In a study assessing gamma-band activity in musically trained children, IGF was shown to be higher over frontal areas, compared to children without musical training [109].

### 5.3. Somatosensory Processing

Recent studies demonstrated the association of IGF with certain aspects of somatosensory processing (Table 8). Spooner et al. [110] reported a significant increase in peak gamma frequency for the second somatosensory stimulus compared to the first one of the pair, pointing to IGF involvement in sensory gating. Interestingly, the elevation was even higher in HIV-infected adults [110]. Similarly, Cheng et al. [111] observed higher IGF for subjects showing more pronounced sensory gating responses in the early P35m component of somatosensory-evoked magnetic fields. On the contrary, in paroxysmal kinesigenic dyskinesia disorder, lower peak gamma frequencies of somatosensory-evoked potentials were discovered compared to healthy subjects [25].

## 6. Peak Gamma Frequency in Motion and Cognitive Processes

The activity within the gamma range is known to be implicated in many processes, including motor commands [112] and cognitive processing [14].

### 6.1. Motor Activity

High-frequency gamma oscillations are involved in movement execution [27]. Some evidence suggests that each individual has a profile of gamma peaks for specific motor outputs that is consistent over time [113]. However, peak gamma frequencies in the primary motor cortex differ depending on the effector: For example, the peak gamma frequency during foot dorsiflexion is significantly lower compared to the peak frequency during elbow flexion [27]. Muthukumaraswamy [28] demonstrated that IGF in the primary motor cortex is higher during the first movement of a sequence in comparison to the peak frequency during the following movements. Zheng [35] showed that peak gamma frequency increased in rats as their speed of running increased. In subjects with paroxysmal kinesigenic dyskinesia, Liu et al. [25] demonstrated a substantially reduced peak frequency compared with healthy controls. Importantly, Heinrichs-Graham et al. [114] showed that the peak frequency of the movement-related gamma synchronization can be modulated by visual distractors: IGF was significantly higher during incongruent conditions than the congruent ones. More details are provided in Table 9.

### 6.2. Cognitive Processes

Kucewicz et al. [115] proposed that there are two distinct gamma oscillatory activity types involved in cognitive processing that can be categorized into narrowband and broadband gamma activities, with the narrowband gamma activity being centered on a specific gamma frequency peak. Indeed, most studies that estimated IGF during cognitive task performance found it to be within the lower gamma range (30–60 Hz) (Table 10). Research in monkeys demonstrated that spatial attention increases peak frequency [36] which also differs depending on where attention is directed: Higher peak frequency was observed when attention was directed to the background, compared to when the foreground was attended [33]. Bosman et al. [116] showed an increase in IGF in the primary visual cortex of monkeys when attention was directed to behaviorally relevant stimuli, compared to irrelevant stimuli. However, human studies showed no evidence of peak gamma frequency modulation in the primary visual cortex due to spatial attention [117] or monitoring of moving stimuli [118]. The alternation rate for bistable images in perceptual rivalry tasks was negatively related to IGF in the primary visual cortex [119]. Chen et al. [29] showed a significant positive correlation between peak gamma frequency and working memory performance. In line with this, some studies reported a positive relationship between IGFs and performance in memory tasks during different stages of sedation with either propofol [79] or isoflurane [78]. Furthermore, patients diagnosed with multiple sclerosis whose gamma peak frequencies were slower tended to perform worse in the neuropsychological tests of verbal memory, attention and executive functions [77]. Supporting results were reported by Insel et al. [37] in rats where IGF negatively correlated with the median decision times. Yet, when the control of age was added, the relationship was non-significant. Finally, the relationship of peak gamma frequencies with language processing was observed by Rufener and Zaehle [5] who found a positive correlation between IGF and phonological awareness in dyslexic patients. Nevertheless, it should be mentioned that a few studies did not observe any relationship between estimated IGFs and performance in complex planning tasks [6] or inhibition interference [7].

## 7. Summary

In the present work, we overviewed the available findings concerning the relationships of IGF with various factors. Even though there is a relatively large number of studies that assessed IGF, only a small fraction considered IGF as a primary measure of interest. The studies included in this review showed that IGFs span a wide range of frequencies (~30–100 Hz). In addition to the inter-individual differences, the wide range of estimated IGFs could be partly due to the variability of methods used to extract these measures. For convenience, the included studies are summarized in Table 11, wherein the number of studies for each IGF estimation modality, task and factor related to IGF is provided. The majority of studies used visual stimulation for IGF estimation, far fewer utilized auditory stimulation, while only a few studies employed somatosensory stimulation or motor and cognitive tasks. However, it is evident that factors related to IGF vary considerably among different stimulation types used for IGF extraction. For example, IGF estimated from the responses to visual stimulation seems to be mainly related to endogenous factors, such as anatomical and neurochemical measures, as well as visual processing, while IGF from the auditory modality was also shown to be linked with cognitive processes. The abovementioned suggests that IGF estimated from different modalities may reflect different functional aspects, being reflective of the activity in the underlying sources. For example, auditory steady-state stimulation results in the activation of both auditory cortices and the frontal region [6,8] potentially being related to gamma implicated in cognitive processing [120]. Conversely, visually evoked or induced gamma activity is generated in the occipital regions [47,50,51] where visual information is processed. Research employing different sensory modalities to estimate IGF and covering several different functional aspects in the same population is required to establish more robust IGF relationships with various processes. In addition, future studies should address the need for the optimization of IGF extraction methods, which could help to estimate the IGF range and correlates more precisely. In general, the present review suggests that IGF seems to reflect certain properties of brain functioning; thus, future studies could potentially provide the basis for the use of IGF as an electrophysiological biomarker.

## Figures and Tables

**Table 1:** Selected studies that reported IGF’s relationship with anatomical factors.

Author and Year	Sample	Methods	IGF Measure	Relevant Findings
Robson et al., 2015 [22]	34 healthy adults	MEG, MRS, visual gratings	Power	IGF: 52.5 ± 4.4 Hz, ~45–65 Hz. No correlation of IGF with V1 surface area and thickness
van Pelt et al., 2018 [23]	158 healthy adults	MEG, MRI, visual gratings	Amplitude	IGF: 56.2 ± 5.4 Hz, 41.5–72.9 Hz. Positive correlation of IGF with occipital thickness (13.4 Hz per mm increase). Negative correlation of IGF with occipital surface area (−0.044 Hz per cm^2^ increase) and pericalcarine volume (−1.88 Hz per cm^3^ increase)
Proskovec et al., 2020 [26]	94 healthy adults	MEG, MRI, electrical stimulation of the right median nerve	Power	IGF: ~30–100 Hz. Negative correlation of IGF with S1 thickness: 27.25 Hz decrease per 1 mm increase
Gregory et al., 2016 [44]	10 healthy adults	MEG, fMRI, visual gratings	Power	IGF: 41.15–70.41 Hz. Positive correlation of IGF with V1 surface area (rho = 0.38)
Pinotsis et al., 2013 [45]	Simulated data	Dynamic causal modeling	Power	IGF: ~45–60 Hz. Positive correlation of IGF with V1 columnar width (r = 0.27)
Schwartzkopf et al., 2012 [46]	16 healthy adults	MEG, MRI, visual gratings	Power	IGF: 44.6–57 Hz. Positive correlation of IGF with V1 (Rs = 0.63) and V2 (Rs = 0.54) surface area. No correlation of IGF with V1 thickness
Gaetz et al., 2012 [47]	46 healthy adults, 13 healthy children	MEG, MRI, visual gratings	Amplitude	IGF: ~35–70 Hz. Positive correlation of IGF with pericalcarine thickness (r^2^ = 0.059), cuneus thickness (r^2^ = 0.115) and cuneus volume (r^2^ = 0.13)
Kienitz et al., 2021 [48]	2 macaque monkeys	In vivo LFP, V1 lesions, visual illusory stimuli	Power	After V1 lesion, IGF decreased by 5.73 ± 0.86 Hz in V4 in one monkey
Perry et al., 2013 [49]	12 healthy adults	MEG, MRI, visual gratings	Amplitude	IGF: ~40–70 Hz. No correlation of IGF with V1 surface area, thickness or volume
Muthukumaraswamy et al., 2010 [50]	30 healthy adults	MEG, MRI, visual gratings	Power	IGF: 51.4 ± 6.6 Hz, 42–64.5 Hz. Positive correlation of IGF with pericalcarine cortical thickness (R = 0.392)
Shaw et al., 2013 [51]	37 healthy adults: 19 remitted depression (RD), 18 never depressed (ND)	MEG, MRS, visual gratings	Power	IGF in RD: 57.64 Hz, IGF in ND: 55.83 Hz, non-significant difference. No correlation of IGF with pericalcarine surface area, positive correlation of IGF with pericalcarine thickness (r = 0.32)
Zaehle et al., 2011 [52]	17 healthy adults	EEG, MRI, visual gratings	Amplitude	IGF: ~20–50 Hz. Positive correlation of IGF with corpus callosum white matter density (r = 0.44–0.65)

Abbreviations: EEG—electroencephalogram; fMRI—functional magnetic resonance imaging; IGF—individual gamma frequency; LFP—local field potential; MEG—magnetoencephalogram; MRS—magnetic resonance spectroscopy; ND—never depressed; RD—remitted depression.

**Table 2:** Selected studies that reported IGF’s relationship with neurochemical factors.

Author and Year	Sample	Methods	IGF Measure	Relevant Findings
Robson et al., 2015 [22]	34 healthy adults	MEG, MRS, visual gratings	Power	IGF: 52.5 ± 4.4 Hz, ~45–65 Hz. No correlation of IGF with V1 surface area and thickness. Positive correlation of IGF with V1 GABA (R = 0.34), but insignificant after including age as a covariate
Wyss et al., 2017 [24]	15 healthy adults	EEG, MRS, auditory stimulation	Power	IGF: ~30–160 Hz. No correlation of IGF with GABA and glutamate
Chen et al., 2014 [29]	12 adults with schizophrenia; 12 healthy adults	EEG, MRS, working memory assessment	Amplitude	IGF: ~30–50 Hz. Positive correlation of IGF with DLPFC GABA (r = 0.58)
Lally et al., 2014 [30]	14 healthy adults	EEG, MRI, categorization task	Power	Negative correlation of IGF with glutamate concentration in the occipital cortex (r = −0.54)
Morgan et al., 2008 [38]	Wistar rats	In vitro LFP, arachidonylcyclopropylamide (ACPA) and LY320135	Power	In hippocampal entorhinal cortex slices, IGF pre-injection: 40.7 ± 2.4 Hz; IGF after CB1R agonist (ACPA) injection decreased to 35.6 ± 1.8 Hz, but returned to 41.2 ± 1.8 Hz after CB1R antagonist (LY320135) injection
Pálhalmi et al., 2004 [39]	Wistar rats	In vitro LFP, carbachol and (RS)-3,5-dihydroxyphenylglycine (DHPG)	Power	IGF post-carbachol: 31.5 ± 0.7 Hz; IGF post-DHPG: 41.2 ± 0.6 Hz. Positive correlation of IGF with DHPG dosage
Shaw et al., 2013 [51]	37 healthy adults: 19 remitted depression (RD), 18 never depressed (ND)	MEG, MRS, visual gratings	Power	IGF in RD: 57.64 Hz, IGF in ND: 55.83 Hz, non-significant difference. No correlation of IGF with occipital GABA
Edden et al., 2009 [57]	13 healthy adults	MEG, MRS, visual gratings	Amplitude	IGF: 50.9 ± 1.3 Hz, 43.5–58.0 Hz. Positive correlation of IGF with V1 GABA concentration (r = 0.67)
Muthukumaraswamy et al., 2009 [58]	12 healthy adults	MEG, MRS, fMRI, visual gratings	Amplitude	IGF: 40–66 Hz. Positive correlation of IGF with V1 GABA concentration (R = 0.68)
Gaetz et al., 2011 [59]	9 healthy adults	MEG, MRI, MRS, visual stimuli and motor responses	Amplitude	IGF: ~70–80 Hz. Positive correlation of IGF with M1 GABA concentration: R^2^ = 0.46 (3.9 Hz increase per 0.1 GABA increase)
Cousijn et al., 2014 [60]	50 healthy adults	MEG, MRS, visual gratings	Power	IGF: ~40–75 Hz. No correlation of IGF with occipital GABA and glutamate
Kujala et al., 2015 [61]	13 healthy adults	MEG, PET, MRI, working memory assessment	Amplitude	IGF: ~40–100 Hz. Positive correlation of IGF with GABA_A_ receptor density in V1 (rho = 0.74)
Campbell et al., 2014 [62]	16 healthy adults	MEG, alcohol, visual gratings, finger movement task	Amplitude	IGF pre-alcohol: ~55 Hz; IGF post-alcohol: ~50 Hz. Drug and time interaction for visual IGF, where IGF decreased after alcohol administration
Shaw et al., 2015 [63]	20 healthy adults	MEG, MRI, visual gratings, ketamine	Amplitude	For high contrast gratings, IGF pre-ketamine: ~51 Hz; IGF post-ketamine: ~49 Hz
Lozano-Soldevilla et al., 2014 [64]	32 healthy adults	MEG, lorazepam, working memory assessment	Power	IGF pre-lorazepam: >75 Hz; IGF post-lorazepam: <75 Hz
Magazzini et al., 2018 [65]	15 healthy adults	MEG, tiagabine, visual gratings	Power	IGF after placebo: ~53 Hz; IGF after tiagabine: ~50 Hz
Kocsis et al., 2014 [66]	Rats	In vivo LFP, D4 receptor agonist A-412997 (Tocris) injections	Power	IGF pre-injection: 51 ± 1 Hz; IGF post-injection: 46 ± 2 Hz
Kühn et al., 2017 [67]	42 Wistar rats	In vivo LFP, levodopa or apomorphine injections, behavioral testing	Power	IGF decreased with apomorphine dosage from ~65 Hz to ~60 Hz in Parkinson’s disease animal model and controls; IGF increased with levodopa dosage from ~60 Hz to ~65 Hz, only in controls
Craig and McBrain 2015 [68]	Nkx2–1-cre:RCE and Htr3a-GFP mice.	In vitro LFP, kainate	Power	Kainate evoked gamma in hippocampal slices. IGF in CA3 region: 52 ± 1.2 Hz, ~40–65 Hz; IGF in CA1 region: 63 ± 0.87 Hz, ~40–80 Hz

Abbreviations: ACPA—arachidonylcyclopropylamide; DHPG—(RS)-3,5-dihydroxyphenylglycine; DLPFC—dorsolateral prefrontal cortex; EEG—electroencephalogram; fMRI—functional magnetic resonance imaging; IGF—individual gamma frequency; LFP—local field potential; MEG—magnetoencephalogram; MRI—magnetic resonance imaging; MRS—magnetic resonance spectroscopy; PET—positron emission tomography.

**Table 3:** Selected studies that reported IGF’s relationship with neuropsychiatric disorders.

Author and Year	Sample	Methods	IGF Measure	Relevant Findings
Rufener et al., 2021 [5]	32 children with developmental dyslexia (DD), 26 healthy children	EEG, auditory steady-state stimulation, phonological awareness task	Power	IGF in DD: 40.63 ± 5.76 Hz; IGF in controls: 45.69 ± 5.85 Hz
Griskova-Bulanova et al., 2020 [9]	18 adults with schizophrenia (SZ), 18 healthy adults	EEG, auditory steady-state stimulation	Phase-locking	IGF in SZ: 44 ± 7 Hz; IGF in controls: 49 ± 8 Hz
Dickinson et al., 2015 [18]	33 healthy adults	EEG, visual gratings, Autism Spectrum Quotient (AQ)	Power	IGF: 59.37 ± 15.66 Hz, 30.27–89.66 Hz. Positive correlation of IGF with AQ score (r = 0.58)
Güntekin et al., 2022 [21]	60 healthy young (HY) adults, 60 healthy elderly (HE), 59 Alzheimer’s patients (AD)	rsEEG	Power	IGF: ~30–43 Hz. IGF in AD: ~33 ± 3 Hz; IGF in HE: ~35 ± 2 Hz; IGF in HY: ~37 ± 1 Hz
Chen et al., 2014 [29]	12 adults with schizophrenia; 12 healthy adults	EEG, MRS, working memory assessment	Amplitude	IGF: ~30–50 Hz. Positive correlation of IGF with prefrontal GABA (r = 0.58)
Dickinson et al., 2016 [72]	28 adults with autism (ASD), 39 healthy adults	EEG, visual gratings	Power	IGF in ASD: 62.19 ± 10.04 Hz; IGF in controls: 51.61 ± 10.75 Hz
Perry et al., 2014 [73]	12 adults with photosensitive epilepsy, 9 with non-photosensitive epilepsy; 12 healthy adults	MEG, visual gratings	Amplitude	No differences between groups
Hepschke et al., 2021 [74]	18 adults with visual snow syndrome (VSS), 16 healthy adults	MEG, MRI, visual gratings	Power	No difference in IGF between groups. IGF in VSS: 53.17 Hz; IGF in controls: 52.63 Hz
Brealy et al., 2015 [75]	15 adults with schizoaffective bipolar disorder (SABP), 22 healthy adults	MEG, visual gratings	Amplitude	IGF in SABP: ~45 Hz transient, ~40 Hz sustained; IGF in controls: ~50 Hz transient, ~45 Hz sustained; no significant difference
Arnfred et al., 2015 [76]	16 adults with schizophrenia spectrum (SZS)	EEG, proprioceptive stimulation, SZS symptoms examination	Amplitude	IGF: ~25–38 Hz. Negative correlation of IGF with SZS symptom scores (r = −0.76)
Arrondo et al., 2009 [77]	27 adults with multiple sclerosis (MS), 22 healthy adults	EEG, auditory steady-state stimulation, cognitive assessment	Amplitude	IGF in cognitively impaired MS: 39.79 Hz; IGF in cognitively unimpaired MS: 43.85 Hz; IGF in controls: 43.84 Hz

Abbreviations: AD—Alzheimer’s disease; ASD—autism spectrum disorder; AQ—Autism Spectrum Quotient; DD—developmental dyslexia; EEG—electroencephalogram; HE—healthy elderly; HY—healthy young; IGF—individual gamma frequency; MEG—magnetoencephalogram; MRI—magnetic resonance imaging; MRS—magnetic resonance spectroscopy; MS—multiple sclerosis; rsEEG—resting-state electroencephalogram; SABP—schizoaffective bipolar disorder; SZ—schizophrenia; SZS—schizophrenia spectrum; VSS—visual snow syndrome.

**Table 5:** Selected studies that reported IGF’s relationship with age.

Author and Year	Sample	Methods	IGF Measure	Relevant Findings
Duygun et al., 2022 [20]	60 healthy young (HY) adults, 60 healthy elderly (HE) and 26 healthy children (HC)	rsEEG	Power	IGF in HC: ~34 Hz; IGF in HY: ~37 Hz; IGF in HE: ~35 Hz
Güntekin et al. [21]	60 healthy young (HY) adults, 60 healthy elderly (HE) and 59 Alzheimer’s patients (AD)	rsEEG	Power	IGF: ~30–43 Hz. IGF in AD: ~33 ± 3 Hz; IGF in HE: ~35 ± 2 Hz; IGF in HY: ~37 ± 1 Hz
Robson et al., 2015 [22]	34 healthy adults	MEG, MRS, visual gratings	Power	IGF: 52.5 ± 4.4 Hz, ~45–65 Hz. Negative correlation of IGF (r = −0.69) with age (M_age_ = 33.7 ± 11.9)
van Pelt et al., 2018 [23]	158 healthy adults	MEG, MRI, visual gratings	Amplitude	IGF: 56.2 ± 5.4 Hz, 41.5–72.9 Hz. Negative correlation of IGF with age (−0.52Hz per year; F age: 23.2 ± 3.9, M_age_: 24.3 ± 5.4)
Wyss et al., 2017 [24]	15 healthy adults	EEG, MRS, auditory stimulation	Power	IGF: ~30–160 Hz. No correlation of IGF with age (19–31 years)
Proskovec et al., 2020 [26]	94 healthy adults	MEG, MRI, electrical stimulation of the right median nerve	Power	No correlation of IGF with age (22–72 years)
Purcell et al., 2004 [31]	38 healthy adults	EEG, auditory steady-state stimulation, gap detection task	Amplitude	IGF in young (18–43 years): 41 ± 5 Hz; IGF in old (60–78 years): 37 ± 4 Hz
Insel et al., 2012 [37]	12 rats	In vivo LFP, decision-making task	Power	IGF: 50–60Hz. IGF in younger rats: 56.4 Hz; IGF in older rats: 53.5 Hz
Schwartzkopf et al., 2012 [46]	16 healthy adults	MEG, MRI, visual gratings	Power	IGF: 44.6–57 Hz. No correlation of IGF with age (19–34 years)
Gaetz et al., 2012 [47]	46 healthy adults, 13 healthy children	MEG, MRI, visual gratings	Amplitude	IGF: ~35–70 Hz. Negative correlation of IGF with age (8.7–45.3 years): r^2^ = 0.46
Muthukumaraswamy et al., 2010 [50]	30 healthy adults	MEG, MRI, visual gratings	Power	IGF: 51.4 ± 6.6 Hz, 42–64.5 Hz. Negative correlation of IGF (r = −0.47) with age (19–44 years)
Shaw et al., 2013 [51]	37 healthy adults: 19 remitted depression (RD), 18 never depressed (ND)	MEG, MRS, visual gratings	Power	IGF in RD: 57.64 Hz, IGF in ND: 55.83 Hz, non-significant difference. No correlation of IGF with age (19–35 years)
Gaetz et al., 2011 [59]	9 healthy adults	MEG, MRI, MRS, visual stimuli and motor responses	Amplitude	IGF: ~70–80 Hz. Negative correlation of IGF with age (22.7–42.7 years): R^2^ = 0.40; 4.8 Hz decrease per 10 years
Kahlbrock et al., 2012 [89]	26 adults with liver cirrhosis, 8 healthy adults	MEG, MRI, selective attention task with visual and auditory stimulation	Power	IGF: ~35–65 Hz. IGF in low age (≤59 years) group: 52 Hz; IGF in high age (>59 years) group: 46 Hz
Murty et al., 2020 [90]	236 healthy elderly, 47 younger adults	EEG, visual gratings	Power	Negative correlation of IGF with age (50–88 years): β = −0.08 for fast gamma; β = −0.16 for slow gamma
Orekhova et al., 2015 [91]	27 healthy children	EEG, MEG, visual gratings	Power	IGF: 50–97.5 Hz. Negative correlation of IGF with age (8–15 years): rho = −0.58–0.8
Orekhova et al., 2018 [92]	27 healthy adults, 50 healthy children	MEG, visual gratings	Power	Negative correlation of IGF with age: −1.71 Hz/year for children, −0.64 Hz/year for adults
Wiesman and Wilson 2019 [93]	77 healthy adults	MEG, MRI, visual grid stimuli	Amplitude	IGF: ~48–70 Hz. Negative correlation of IGF (r = −0.29) with age (22–72 years)
Stroganova et al., 2015 [94]	21 children with autism (ASD), 26 healthy children	MEG, visual gratings	Power	IGF: 57.5–92.3 Hz. Negative correlation of IGF with age (7–15 years), rho = −0.6. Positive correlation of IGF modulation with age in healthy group (r = 0.45), but not ASD group
Poulsen et al., 2007 [95]	33 healthy adults	EEG, auditory steady-state stimulation	Amplitude	IGF: 41 ± 4.7 Hz, 32–52 Hz. Positive correlation of IGF with age (19–45 years): 38 Hz at 19 years, 46 Hz at 45 years
Poulsen et al., 2009 [96]	65 healthy children, 23 healthy adults	EEG, auditory steady-state stimulation	Amplitude	IGF at 10 years: 35.3 ± 5.76 Hz, 25–52 Hz; IGF at 11.5 years: 36.5 ± 5.55 Hz, 27–55 Hz; IGF in adults (19–45 years): 41.2 ± 4.7 Hz

Abbreviations: AD—Alzheimer’s disease; ASD—autism spectrum disorder; EEG—electroencephalogram; HC—healthy children; HE—healthy elderly; HY—healthy adults; IGF—individual gamma frequency; MEG—magnetoencephalogram; MRI—magnetic resonance imaging; MRS—magnetic resonance spectroscopy; ND—never depressed; RD—remitted depression; rsEEG—resting-state electroencephalogram.

**Table 6:** Selected studies that reported IGF’s relationship with visual processing.

Author and Year	Sample	Methods	IGF Measure	Relevant Findings
van Pelt and Fries 2013 [19]	14 healthy adults	MEG, visual gratings	Power	Negative correlation of IGF with stimulus eccentricity: −0.91 Hz per 1-degree increase for moving stimulus, −0.95 Hz per 1-degree increase for stationary stimulus. Negative correlation of IGF with stimulus size: −0.69 Hz per 1-degree increase in diameter
van Pelt et al., 2018 [23]	158 healthy adults	MEG, MRI, visual gratings	Amplitude	IGF: 56.2 ± 5.4 Hz, 41.5–72.9 Hz. IGF depends on stimulus contrast. IGF for high contrast: 56.3Hz; IGF for low contrast: 52.4 Hz (0.078 Hz per 1% increase in stimulus contrast). IGF for high velocity: 56.2 Hz; IGF for low velocity: 52.4 Hz; IGF for stationary: 50 Hz (7.2 Hz per 1 deg/s increase in velocity)
Jia et al., 2013 [32]	7 macaque monkeys	In vivo LFP, visual gratings	Power, phase-locking	IGF for small stimuli: 43 Hz; IGF for large stimuli: 37 Hz. IGF for small gratings: 47 Hz; IGF for large gratings: 38 Hz
Lima et al., 2010 [33]	4 rhesus monkeys	In vivo LFP, visual gratings and plaids	Power	IGF increased from 58 to 68 Hz with stimulus luminance or contrast increase. IGF for central regions: 60 Hz (gratings) and 73 Hz (plaids); IGF for peripheral regions: 47 Hz (gratings) and 58 Hz (plaids)
Das and Ray 2018 [36]	2 rhesus monkeys	In vivo LFP, visual attention task	Power	IGF increased with stimulus contrast. IGF for low contrast: 40 Hz; IGF for medium contrast: 44–48 Hz; IGF for high contrast: 56 Hz
Gregory et al., 2016 [44]	10 healthy adults	MEG, fMRI, visual gratings	Power	IGF: 41.15–70.41 Hz. IGF depends on stimulus eccentricity, but not size. IGF for central stimuli: 54.73 ± 6.87 Hz (small size) and 55.4 ± 8.27 Hz (large size); IGF for peripheral stimuli: 59.89 ± 6.05 Hz (small size), 60.19 ± 6.68 Hz (large size)
Perry et al., 2013 [49]	12 healthy adults	MEG, MRI, visual gratings	Amplitude	IGF: ~40–70 Hz. No difference in IGF across different stimulus sizes
Perry et al., 2014 [73]	12 adults with photosensitive epilepsy, 9 with non-photosensitive epilepsy; 12 healthy adults	MEG, visual gratings	Amplitude	IGF for low stimulus contrast: ~47 Hz; IGF for high stimulus contrast: ~57 Hz
Kahlbrock et al., 2012 [89]	26 adults with liver cirrhosis, 8 healthy adults	MEG, MRI, selective attention task with visual and auditory stimulation	Power	IGF: ~35–65 Hz. IGF in high critical flicker frequency (CFF) group: 51 Hz; IGF in low CFF group: 45.9 Hz
Orekhova et al., 2015 [91]	27 healthy children	EEG, MEG, visual gratings	Power	IGF: 50–97.5 Hz. IGF for slow velocity: 50–67.5 Hz; IGF for medium velocity: 77.5–82.5 Hz; IGF for high velocity: 95–97.5 Hz
Orekhova et al., 2018 [92]	27 healthy adults, 50 healthy children	MEG, visual gratings	Power	IGF increased from low to high stimulus velocity by 15.3 Hz for children (66.1 ± 6.1 Hz to 82.2 ± 10.8 Hz), by 14.6 Hz for adults (55.7 ± 5.7 Hz to 70 ± 8.5 Hz)
Stroganova et al., 2015 [94]	21 children with autism (ASD), 26 healthy children	MEG, visual gratings	Power	IGF: 57.5–92.3 Hz. Reduced IGF modulation due to stimulus velocity for ASD group vs. healthy. IGF for low velocity: ~65 Hz; IGF for high velocity: ~85 Hz
Hadjipapas et al., 2015 [97]	9 healthy adult humans, 2 rhesus monkeys	MEG, in vivo LFP, visual gratings	Power	IGF increased by ~19 Hz (from ~26 Hz to ~45 Hz) in monkeys, by ~8 Hz (from ~38 Hz to ~46 Hz) in humans after increasing stimulus contrast
Krishnakumaran et al., 2022 [98]	2 macaque monkeys	In vivo LFP, visual gratings	Power	IGF increased due to stimulus contrast. IGF for low contrast: ~35 Hz; IGF for high contrast: ~50 Hz. IGF decreased due to stimulus size. IGF for small stimulus: ~50 Hz; IGF for big stimulus: ~45 Hz.
Perry et al., 2015 [99]	12 healthy adults	MEG, visual gratings and plaids	Amplitude	IGF for low stimulus contrast: 49 Hz; IGF for high stimulus contrast: 60 Hz. IGF for plaid stimuli: ~60 Hz; IGF for gratings: ~45–50 Hz
Ray and Maunsell 2010 [100]	2 rhesus monkeys	In vivo LFP, visual gratings	Power	IGF increased by 6.8 Hz with double increase in stimulus contrast. IGF for 25% contrast: 37–38 Hz; IGF for 100% contrast: 52–53 Hz
Roberts et al., 2013 [101]	2 macaque monkeys	In vivo LFP, visual gratings	Power, phase-locking	IGF for low stimulus contrast: ~20 Hz; IGF for high stimulus contrast: ~45 Hz
Swettenham et al., 2009 [102]	15 healthy adults	MEG, visual gratings	Power	IGF for stationary stimuli: 43.5 ± 9 Hz, 27–55.5 Hz; IGF for moving stimuli: 51 ± 7.7 Hz, 40–60 Hz
Murty et al., 2018 [103]	2 bonnet monkeys, 19 healthy adult humans	In vivo LFP, EEG, visual gratings	Power	In monkeys, IGF depends on stimulus orientation. IGF for 90° orientation: 58 ± 0 Hz (monkey 1) and 55.65 ± 0.21 Hz (monkey 2); IGF for 45° orientation: 51.27 ± 0.36 Hz (monkey 1) and 52.29 ± 0.48 Hz (monkey 2). IGF increased with higher stimulus contrast in monkeys (by 3.3–9.6 Hz), but not in humans
Shirhatti et al., 2022 [104]	2 bonnet monkeys	In vivo LFP, visual gratings	Power	IGF increased due to annular cut, orientation and phase discontinuities in grated stimuli
Gieselmann and Thiele 2008 [105]	2 macaque monkeys	In vivo LFP, visual gratings	Power	IGF decreased by 2.95 Hz (monkey 1) or 1.58 Hz (monkey 2) for every degree increment in stimulus size
Stauch et al., 2021 [106]	30 healthy adults	MEG, visual gratings	Power	In a sequence of the same repeated stimulus, IGF did not change in the first 10 repetitions, but with further repetitions increased gradually by 0.05Hz/repetition or 6 Hz increase over 120 repetitions
Peter et al., 2021 [107]	4/2 monkeys	In vivo LFP, visual gratings, natural images	Power	IGF was specific to stimulus. IGF decreased for early trials (~45 Hz), but increased for later trials (~47 Hz)
Brunet et al., 2015 [108]	2 macaque monkeys	In vivo LFP, natural images	Power	IGF before saccade: 50–80 Hz; IGF immediately after saccade: 30–40 Hz

Abbreviations: ASD—autism spectrum disorder; EEG—electroencephalogram; fMRI—functional magnetic resonance imaging; IGF—individual gamma frequency; LFP—local field potential; MEG—magnetoencephalogram; MRI—magnetic resonance imaging.

**Table 7:** Selected studies that reported IGF’s relationship with auditory processing.

Author and Year	Sample	Methods	IGF Measure	Relevant Findings
Purcell et al., 2004 [31]	38 healthy adults	EEG, auditory steady-state stimulation, gap detection task	Amplitude	IGF in young (18–43): 41 ± 5 Hz; IGF in old (60–78): 37 ± 4 Hz. Positive correlation of IGF with frequency modulation detection (r = 0.72), negative correlation with gap detection latency (r = −0.43)
Baltus and Herrmann 2015 [41]	35 healthy adults	EEG, auditory steady-state stimulation, gap detection task	Amplitude	IGF: 46.5 ± 6.38 Hz. Negative correlation of IGF with gap detection threshold (r = −0.46)
Baltus et al., 2020 [83]	16 healthy adults	EEG, tACS, auditory steady-state stimulation, gap detection task	Amplitude	IGF: ~35–60 Hz. Negative correlation of IGF with gap detection threshold (rho = −0.6). Positive correlation of IGF with change in performance after tACS in the experimental group (rho = 0.81), but not in the control group
da Silva et al., 2021 [109]	31 healthy children: 16 with musical training, 15 without musical training	EEG, motor and music-related tasks	Power	Over F3-F4 channels, IGF in musically trained: 35 Hz; IGF in musically untrained: 33 Hz

Abbreviations: EEG—electroencephalogram; IGF—individual gamma frequency; tACS—transcranial alternating current stimulation.

**Table 8:** Selected studies that reported IGF’s relationship with somatosensory processing.

Author and Year	Sample	Methods	IGF Measure	Relevant Findings
Liu et al., 2018 [25]	19 adults with paroxysmal kinesigenic dyskinesia (PKD), 18 healthy adults	MEG, genetic analysis, electric stimulation of the wrist	Power	IGF in PKD: ~40 Hz, ~30–50 Hz; IGF in controls: ~60 Hz, ~45–90 Hz. Lower IGF in PRRT2 gene-related PKD (~35 Hz) vs. non-PRRT2 PKD (~44 Hz)
Spooner et al., 2018 [110]	43 HIV-infected, 28 healthy adults	MEG, electrical stimulation of the right median nerve	Power	IGF: ~30–90 Hz. Higher IGF for second stimulus vs. first stimulus in a sequence, stronger effect in HIV group
Cheng et al., 2016 [111]	22 healthy adults	MEG, somatosensory and auditory Go–Nogo tasks	Power	IGF: ~73 Hz, 40–89 Hz. Negative correlation of IGF with the ratio of responses to the second stimulus vs. the first stimulus (r = −0.57) in P35m component of somatosensory-evoked magnetic fields

Abbreviations: IGF—individual gamma frequency; MEG—magnetoencephalogram; PKD—paroxysmal kinesigenic dyskinesia.

**Table 9:** Selected studies that reported IGF’s relationship with motor activity.

Author and Year	Sample	Methods	IGF Measure	Relevant Findings
Liu et al., 2018 [25]	19 adults with paroxysmal kinesigenic dyskinesia (PKD), 18 healthy adults	MEG, genetic analysis, electric stimulation of the wrist	Power	IGF in PKD: ~40 Hz, ~30–50 Hz; IGF in controls: ~60 Hz, ~45–90 Hz. Lower IGF in PRRT2 gene-related PKD (~35 Hz) vs. non-PRRT2 PKD (~44 Hz)
Cheyne et al., 2008 [27]	9 healthy adults	MEG, finger, bicep, foot movements	Power	IGF: 66–85 Hz. IGF for index finger abduction: 75.3 Hz; IGF for foot dorsiflexion: 67.4 Hz; IGF for bicep contraction: 73.9 Hz
Muthukumaraswamy 2010 [28]	19 healthy adults	MEG, movements of index finger, first dorsal interosseous muscle contractions	Power	IGF: 78.2 Hz, 73.5–81 Hz. In a sequence of repetitive movements, higher IGF for initial finger movements compared to later movements
Zheng et al., 2015 [35]	8 Long Evans rats	In vivo LFP, running task	Power	IGF ~30–100 Hz. In the hippocampus, IGF for 3 cm/s running speed: ~60–80 Hz; IGF for 96 cm/s running speed: ~80–100 Hz in the hippocampus
Heinrichs-Graham et al., 2018 [114]	42 healthy adults	MEG, MRI, response inhibition task	Power	IGF: ~60–90 Hz. IGF for incongruent condition: ~75 Hz; IGF for congruent condition: ~70 Hz

Abbreviations: IGF—individual gamma frequency; LFP—local field potential; MEG—magnetoencephalogram; MRI—magnetic resonance imaging; PKD—paroxysmal kinesigenic dyskinesia.

**Table 10:** Selected studies that reported IGF’s relationship with cognitive processes.

Author and Year	Sample	Methods	IGF Measure	Relevant Findings
Rufener et al., 2021 [5]	32 children with developmental dyslexia (DD), 26 healthy children	EEG, auditory steady-state stimulation, phonological awareness task	Power	IGF in DD: 40.63 ± 5.76 Hz; IGF in controls: 45.69 ± 5.85 Hz. Positive correlation of IGF with phonological awareness (r = 0.33) and writing skills (r = 0.37)
Parciauskaite et al., 2021 [6]	37 healthy adults	EEG, auditory steady-state stimulation, cognitive tasks	Power, phase-locking	IGF 35–53 Hz, mostly 41–42 Hz. No correlation of IGF with complex cognitive task performance
Griškova-Bulanova et al., 2022 [7]	70 healthy adults	EEG, auditory steady-state stimulation, cognitive inhibition task	Power, phase-locking	IGF: 40 Hz (phase-locking) and 42 Hz (power), 32–59 Hz. No correlation of IGF with any behavioral measures of cognitive performance
Chen et al., 2014 [29]	12 adults with schizophrenia; 12 healthy adults	EEG, MRS, working memory assessment	Amplitude	IGF: ~30–50 Hz. Positive correlation of IGF with working memory performance (r = 0.59)
Lima et al., 2010 [33]	4 rhesus monkeys	In vivo LFP, visual gratings and plaids	Power	IGF during attention to foreground: ~70 Hz; IGF during attention to background: ~75 Hz
Das and Ray 2018 [36]	2 rhesus monkeys	In vivo LFP, visual attention task	Power	IGF increased by ~2 Hz for low contrast stimuli due to spatial attention
Insel et al., 2012 [37]	12 rats	In vivo LFP, decision-making task	Power	IGF: 50–60Hz. Negative correlation of IGF with median decision time (r = −0.58); non-significant when controlling for age
Arrondo et al., 2009 [77]	27 adults with multiple sclerosis (MS), 22 healthy adults	EEG, auditory steady-state stimulation, cognitive assessment	Amplitude	IGF in cognitively impaired MS: 39.79 Hz; IGF in cognitively unimpaired MS: 43.85 Hz; IGF in controls: 43.84 Hz. Positive correlation of IGF with verbal memory, attention, executive functions and verbal fluency (r = 0.44–0.59)
Munglani et al. 1993 [78]	7 healthy adults	EEG, isoflurane, auditory click stimulation, cognitive tests	Power	IGF: 32.8 Hz, 28–41 Hz. Positive correlation of IGF with performance in within-list recognition and category recognition tasks
Andrade et al. 1996 [79]	12 healthy adults	EEG, propofol, auditory click stimulation, cognitive assessment	Power	IGF: 37.6 Hz, 33.5–41.5 Hz. Positive correlation of IGF with within-list recognition task performance (r = 0.47)
Bosman et al., 2012 [116]	2 monkeys	In vivo LFP, visual gratings	Power, phase-locking	IGF increased by 2–3 Hz when attending relevant vs. irrelevant stimuli
Magazzini et al., 2018 [117]	20 healthy adults	MEG, visual gratings	Power	IGF ~42–65 Hz. No difference in IGF between attend stimulus condition (51.3 Hz) and ignore stimulus condition (50.5 Hz)
Kennedy et al., 2011 [118]	15 healthy adults	MEG, eye-tracking, manual and visual tracking task	Power	IGF: 68.6 Hz, 63–74.5 Hz. No difference in IGF between fixate (at central crosshair) and pursue (target stimulus) conditions
Fesi and Mendola 2015 [119]	12 healthy adults	MEG, perceptual rivalry task	Power	IGF: 62.58 Hz (left hemisphere), 57.77 Hz (right hemisphere), ~30–90 Hz. Negative correlation of IGF in V1 with perceptual rivalry switch rate (r = 0.62 to −0.76)

Abbreviations: EEG—electroencephalogram; DD—developmental dyslexia; IGF—individual gamma frequency; LFP—local field potential; MEG—magnetoencephalogram; MRS—magnetic resonance spectroscopy; MS—multiple sclerosis.

**Table 11:** A summary of selected human and animal studies. The number of studies was counted for each IGF estimation modality, specific tasks within the modalities and the factors related to IGF. In addition, the IGF range within each modality is presented. Factors related to IGF include only those studies that found a statistically significant relationship.

Human Studies				
IGF Estimation Modality	Number of Studies	Stimuli and Tasks Used for IGF Estimation	IGF Range	Factors Related to IGF
Resting-state	2	-	30–43 Hz	Neuropsychiatric disorders: 1 Age: 2
Visual	39	Visual gratings: 37 Visual checkerboard stimulus: 1 Visual plaids: 1 Visual grid stimulus: 1	35–100 Hz	Anatomical: 7 Neurochemical: 7 Neuropsychiatric disorders: 2 Brain states: 1 Age: 8 Visual processing: 15
Auditory	17	Auditory steady-state stimulation: 14 Auditory stimulation with changing intensity: 2 Auditory sensory-gating stimulation: 1 Auditory oddball stimulation: 1	28–63 Hz *	Neuropsychiatric disorders: 4 Brain states: 4 Age: 4 Auditory processing: 4 Cognitive processing: 5
Somatosensory	5	Electrical stimulation of the wrist: 3 Somatosensory Go–Nogo task: 1 Proprioceptive stimulation: 1	25–90 Hz	Anatomical: 1 Neuropsychiatric disorders: 1 Somatosensory processing: 3
Motor	6	Finger/palm muscle contractions: 3 Arm movements: 1 Foot movements: 1 Block movement task: 1 Button press: 1 Manual tracking task: 1	63–85 Hz	Neurochemical: 1 Age: 1 Motor activity: 2 Cognitive processing: 1
Cognitive	8	Working memory tasks: 3 Categorization task: 1 Perceptual rivalry task: 1 Response inhibition task: 1 Selective attention task: 1 Musical performance tasks: 1	40–100 Hz	Neurochemical: 4 Age: 1 Visual processing: 1 Cognitive processing: 4
**In Vivo Animal Studies**				
**IGF Estimation Modality**	**Number of Studies**	**Stimuli and Tasks Used for IGF Estimation**	**IGF Range**	**Factors Related to IGF**
Visual	11	Visual gratings: 10 Natural images: 2 Visual plaids: 1 Visual illusory stimuli: 1	30–80 Hz	Anatomical: 1 Brain states: 1 Visual processing: 9 Cognitive processing: 1
Motor	3	Free movement: 2 Motor tasks: 1	30–100 Hz	Neurochemical: 2 Motor activity: 1
Cognitive	3	Decision-making tasks: 1 Visual attention task: 1 Surface food test: 1 Novel object recognition test: 1	40–60 Hz	Brain states: 1 Age: 1 Visual processing: 1

Abbreviation: IGF—individual gamma frequency. * Wyss et al. [24] reported the IGF range extracted from auditory stimulation to be between 30 and 160 Hz; however, in the other studies, the highest IGF values were up to around 63 Hz.

## Data Availability

Not applicable.
